# Abnormally activated one-carbon metabolic pathway is associated with mtDNA hypermethylation and mitochondrial malfunction in the oocytes of polycystic gilt ovaries

**DOI:** 10.1038/srep19436

**Published:** 2016-01-13

**Authors:** Longfei Jia, Juan Li, Bin He, Yimin Jia, Yingjie Niu, Chenfei Wang, Ruqian Zhao

**Affiliations:** 1 Key Laboratory of Animal Physiology & Biochemistry, College of Veterinary Medicine, Nanjing Agricultural University, Nanjing 210095, P. R. China; 2 College of Animal Science and Technology, Nanjing Agricultural University, Nanjing 210095, P. R. China; 3 Jiangsu Collaborative Innovation Center of Meat Production and Processing, Quality and Safety Control, Nanjing 210095, P. R. China

## Abstract

Polycystic ovarian syndrome (PCOS) is associated with hyperhomocysteinemia and polycystic ovaries (PCO) usually produce oocytes of poor quality. However, the intracellular mechanism linking hyperhomocysteinemia and oocyte quality remains elusive. In this study, the quality of the oocytes isolated from healthy and polycystic gilt ovaries was evaluated *in vitro* in association with one-carbon metabolism, mitochondrial DNA (mtDNA) methylation, and mitochondrial function. PCO oocytes demonstrated impaired polar body extrusion, and significantly decreased cleavage and blastocyst rates. The mitochondrial distribution was disrupted in PCO oocytes, together with decreased mitochondrial membrane potential and deformed mitochondrial structure. The mtDNA copy number and the expression of mtDNA-encoded genes were significantly lower in PCO oocytes. Homocysteine concentration in follicular fluid was significantly higher in PCO group, which was associated with significantly up-regulated one-carbon metabolic enzymes betaine homocysteine methyltransferase (BHMT), glycine N-methyltransferase (GNMT) and the DNA methyltransferase DNMT1. Moreover, mtDNA sequences coding for *12S*, *16S rRNA* and *ND4,* as well as the D-loop region were significantly hypermethylated in PCO oocytes. These results indicate that an abnormal activation of one-carbon metabolism and hypermethylation of mtDNA may contribute, largely, to the mitochondrial malfunction and decreased quality of PCO-derived oocytes in gilts.

Polycystic ovarian syndrome (PCOS) is a common reproductive and endocrine disorder in women of reproductive age^1^, characterized by anovulation, irregular menses, and androgen excess^2^. PCOS patients often produce oocytes of poor quality, leading to lower fertilization^3^,^4^,^5^,^6^,^7^, cleavage and implantation rate^8^, and higher miscarriage rate^9^,^10^. PCOS is closely associated with a number of metabolic aberrations, including dyslipidemia^11^, insulin resistance^12^, type II diabetes mellitus^13^ and hypertension^14^. Interestingly, many metabolic disorders, including cardiovascular diseases^15^ and osteoporotic fractures^16^, are complicated with hyperhomocysteinemia, a condition characterized by an abnormally high level of homocysteine (Hcy) in the blood. Hcy is an important intermediate metabolite in the one-carbon metabolism pathway that contributes to epigenetic gene regulation by donating methyl groups for DNA and/or protein methylation^17^. Elevated serum Hcy levels are reported in PCOS patients^18^,^19^,^20^. Moreover, abnormally high level of Hcy is detected in the follicular fluid of polycystic ovaries (PCO)^21^, in association with poor oocyte quality. Nevertheless, the mechanisms linking high Hcy level and poor oocyte quality remain unclear.

Oocytes are greatly enriched in the number of mitochondria which play critical roles during oocyte maturation^22^,^23^ and fertilization, as well as the initiation and progression of preimplantation development^24^. The primary function of the mitochondrion is ATP production via the oxidative phosphorylation (OXPHOS) pathway. Mammalian mitochondrial DNA (mtDNA) is approximately 16 kb in size, encoding 13 proteins which are subunits of the OXPHOS complexes, 22 transfer RNAs, and 2 ribosomal RNAs^25^. Similar to nuclear DNA, mtDNA is also subjected to epigenetic modifications that influence mitochondrial gene expression, biogenesis and function^26^,^27^. Recently, mtDNA methylation has received increasing attention as changes in mtDNA methylation are found to be associated with mitochondria dysfunction and thereby the pathogenesis of many metabolic diseases^28^,^29^. The methylation status of mtDNA changes in disease development^30^,^31^, environment exposure^32^,^33^,^34^, drug treatment^35^ and aging^36^. Methylation of nuclear DNA is closely related to one-carbon metabolism^37^,^38^,^39^,^40^, yet no evidences are available linking one-carbon metabolism with mtDNA methylation and mitochondrial functions.

In this study, we use polycystic ovaries of gilts as model to investigate the possible association between one-carbon metabolic pathway, mtDNA methylation, mitochondrial function and oocyte quality. The findings will help to understand the underlying causes of poor oocyte quality in PCOS patients.

## Materials and Methods

The experimental protocol was approved by the Animal Ethics Committee of Nanjing Agricultural University, with the project number 2014CB138502. The slaughter and sampling procedures complied with the “Guidelines on Ethical Treatment of Experimental Animals” (2006) No. 398 set by the Ministry of Science and Technology, China.

### Oocyte retrieval and *in vitro* maturation of oocytes

Healthy and polycystic ovaries were obtained from the cross-bred prepubertal gilts (Landrace × Large White × Duroc; 135–170 days of age; 70–120 kg of body weight) slaughtered in a local abattoir. Ovaries in the follicular phase of the ovarian cycle were selected and classified into two groups: healthy (control) and polycystic (PCO). Healthy ovaries contain follicles of approximately 8–10 mm in size with intact and well organized granulosa layers. Polycystic ovaries are defined when follicles were >21 mm in size with smooth and thin walls filled with fluid, and were absent of corpora lutea^41^. Among 2228 ovaries collected and inspected, 147 ovaries were polycystic, comprising 6.6% of the population (Figure S1). Ovaries were kept in 0.9% saline (w/v) supplemented with 75 μg/mL potassium penicillin G and 50 μg/mL streptomycin sulfate at 37 °C and transported to the laboratory within 3 h. *In vitro* oocyte maturation was performed as described earlier^42^. Briefly, cumulus-oocyte complexes (COCs) were aspirated from follicles of ovaries with an 18-gauge needle connected to a 20 mL disposable syringe. After washing three times in HEPES-buffered Tissue Culture Medium 199 (TCM-199) plus 0.8 mmol/L L-Glutamine, and 2% (v/v) cattle serum, the COCs were cultured in groups of 50–60 in 4-well dishes with TCM-199 supplemented with 10% (v/v) cattle serum, 10% (v/v) porcine follicular fluid, 0.8 mmol/L L-Glutamine, 75 μg/mL potassium penicillin G, 50 μg/mL streptomycin sulfate, 15 IU/mL pregnant mare’s serum gonadotropin and 15 IU/mL human chorionic gonadotropin at 38.5 °C with 5% CO_2_, 20% O_2 _for 42–44 h with maximum humidity.

### Determination of Hcy concentration in follicular fluid

Follicular fluid from each ovary was aspirated and pooled as one sample (12 samples per group). Follicle aspirates contaminated with blood were discarded. Follicular fluid was centrifuged in 2000 g for 10 min and the supernatant was collected and frozen at −80 °C until assayed. Hcy level of the collected follicular fluid samples were determined by an automated chemiluminescence technique (Hitachi 7020 Automatic Analyzer, Japan) using a Homocysteine Assay Kit by Enzymatic Cycling Method (Sichuan Maker Biotechnology Co., Ltd., China).

### Developmental potential of oocytes after parthenogenetic activation (PA)

Oocytes with intact cytoplasm and the first polar body were equilibrated in activation solution consisting of 0.3 mol/L mannitol, 0.1 mmol/L MgSO_4_, 0.1 mmol/L CaCl_2_ and 0.01% polyvinyl alcohol and were electrically activated in a 0.5 mm microslide fusion chamber (BTX, USA) by a single direct current pulse of 0.86 kV/cm for 80 μs. After chemical activation with 10 μg/mL cycloheximide and 5 μg/mL cytochalasin B in PZM-3 medium^43^ for 4 h, oocytes were cultured in PZM-3 medium at 38.5 °C in 5% CO_2_, 20% O_2_ with maximum humidity. To assess the *in vitro* developmental ability, percentage of embryos at the 2-cell (24 h after PA), 4-cell (36 h after PA) and blastocyst stages (144 h after PA) was determined. To evaluate the total cell number per blastocyst, blastocysts were randomly collected and stained with 10 μg/mL of Hoechst 33342 in phosphate buffered saline (PBS) for 20 min. The morphology of blastocysts was observed and the total cell number per blastocyst was counted under the epifluorescent microscope with UV-2A filter (Leica DMIRB, UK).

### Determination of relative mtDNA copy number

Fifty oocytes were pooled as one sample (4 samples per group). Total genomic DNA was isolated from pooled oocyte samples and the mtDNA copy number was determined using real-time PCR as previously described with some modifications^44^. For DNA extraction, oocytes were incubated in a lysis solution containing 0.5 mol/L of EDTA, pH 8.0 and 2 mg/mL of proteinase K (Amresco, USA) at 37 °C for 50 min. Primers specific for the coding region of mitochondrial DNA were used for the quantification of the mtDNA copy number, whereas primers specific for the 18S nuclear gene were used for standardization (Table S1). Relative mtDNA copy number was calculated with 2^−△△Ct^ method.

### RNA extraction, reverse transcription and real-time quantitative PCR

To investigate the abundance of mRNA in oocytes, 5–10 oocytes were pooled. Messenger RNA extraction and the reverse transcription were conducted using SuperScript^TM^ III First-Strand Synthesis System (Invitrogen, USA), according to the manufacturer’s protocol. The synthesized cDNA was used for quantitative real-time PCR. 18S was chosen as internal control, and the primer sequences are shown in Table S1. Real-time PCR was performed with Mx3000P (Stratagene, USA). The 2^−△△Ct^ method was used to analyze real-time PCR data.

### Analysis of mitochondrial distribution

Oocytes (30 per group) were stained with 200 nmol/L MitoTracker Red CMXRos (Molecular Probes, USA) for 45 min at 38.5 °C under 5% CO_2_, followed by washing in TCM-199 twice, and then were fixed with 4% paraformaldehyde in PBS for 15 min at room temperature. After three washes with TCM-199 for 20 min, the oocytes were mounted on glass slides and then observed with a laser-scanning confocal microscope (Zeiss LSM 710 META, Germany).

### Analysis of mitochondrial activity

Oocytes were stained with JC-1 (Molecular Probes, USA), a fluorescent dye that accumulates in mitochondria and indicates the membrane potential across the matrix membrane^45^. JC-1 fluorescence has two emission peaks, with the red fluorescence (JC-1 aggregate) indicating high-polarized mitochondria (high membrane potential) and the green fluorescence (JC-1 monomers) indicating low-polarized mitochondria (low membrane potential). Mitochondrial activity can be evaluated by the ratio of the red/green fluorescence intensity^46^. Briefly, denuded oocytes (30 per group) were incubated in JC-1 (10 μmol/L in PBS supplemented with 10% bovine serum albumin) at 37 °C for 30 min, and washed twice in PBS. Stained oocytes were transferred and mounted on glass slides, and then observed with a laser-scanning confocal microscope (Zeiss LSM 710 META, Germany).

### Electron microscope observation of mitochondria

The normal and polycystic follicles from healthy and polycystic ovaries were dissected, respectively, and fixed with 2.5% (v/v) glutaraldehyde in PBS and post-fixed with 1.0% (w/v) osmium tetroxide in the same buffer, followed by dehydration in a graded series of ethanol, propylene oxide treatment, and then embedded in epoxy resin, and sectioned. The ultrathin sections were contrasted with ethanolic uranyl acetate and lead citrate and observed under a transmission electron microscope (JEOL JEM-1210, Japan).

### Immunolabeling and detection of DNA methyltransferase 1 (DNMT1) in oocytes

Oocytes were fixed in 4% paraformaldehyde in PBS for 30 min at room temperature. Oocytes were then transferred to a membrane permeabilization solution (1% Triton X-100 in PBS) for 8–12 h at 4 °C. After 1 hr in blocking buffer (1% bovine serum albumin in PBS), oocytes were subsequently incubated overnight at 4 °C with Anti-DNMT1 antibody (24206-1-AP, Proteintech, USA, diluted 1:200). After three washes (2 min each) in wash buffer (0.1% Tween 20 and 0.01% Triton X-100 in PBS), oocytes were incubated with Alexa Fluor 488 secondary antibody (A-11008, Molecular Probes, USA, diluted 1:200) for 1 h at 38.5 °C under 5% CO_2_. Oocytes were then co-stained with 200 nmol/L MitoTracker Red CMXRos (Molecular Probes, USA) for 45 min at 38.5 °C under 5% CO_2_, followed by washing twice in TCM-199. Finally, the nuclei were counterstained with Hoechst 33342 (10 mg/mL in PBS) for 10 min. The oocytes were mounted on glass slides, and then examined with a confocal laser-scanning microscope (Zeiss LSM 700 META, Germany).

### Protein Extraction and Western Blot Analysis

For protein extraction, 100 oocytes were pooled as one sample (4 samples per group) and lysed in Laemmli sample buffer (SDS sample buffer with 2-mercaptoethanol), and boiled at 100 °C for 15 min. Proteins were subjected to 10% SDS-polyacrylamide gel electrophoresis (PAGE). Western blot analysis for betaine homocysteine methyltransferase (BHMT, 15965-1-AP, Proteintech, USA, diluted 1:500), glycine N-methyltransferase (GNMT, 18790-1-AP, Proteintech, USA, diluted 1:500) and DNMT1 (24206-1-AP, Proteintech, USA, diluted 1:1000) was carried out according to the recommended protocols provided by the manufacturers. The β-actin (AP0060, Bioworld, USA, diluted 1:10,000) was used as loading control in the Western blot analysis. Images were captured by VersaDoc 4000MP system (Bio-Rad, USA) and the band density was analyzed with Quantity One software (Bio-Rad, USA).

### Bisulfite sequencing and PCR amplification

A total of approximately 120 oocytes per group were used for mtDNA methylation analysis. Oocytes were incubated with lysis solution (0.5 mol/L EDTA, pH 8.0; 2 mg/mL proteinase K, Amresco, USA) for 50 min at 37 °C and followed by bisulfite treatment using the EZ DNA Methylation-Direct^TM^ Kit (Zymo Research, USA) according to the manufacturer’s instructions. Modified DNA was then used as a template in PCR amplification with the primers listed in Table S1. The PCR products were cloned to T vector and sequenced (Invitrogen, USA).

### Statistical Analysis

The results are presented as means ± SEM. Comparisons were performed using independent-samples T-Test with SPSS 20.0. Chi-square test was used to evaluate the difference in mitochondrial distribution and methylation status between two groups. The differences were considered statistically significant when *P* < 0.05.

## Results

### Oocytes harvested from PCO show impaired developmental potential

**Figure 1:**
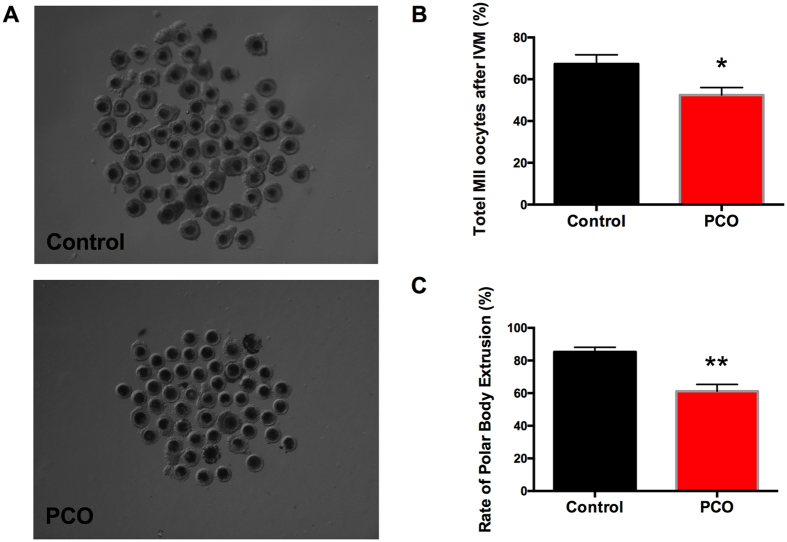
Quality analysis of oocytes from polycystic ovaries of gilts. (**A**) Representative images of porcine COCs (magnification, × 56); (**B**) Number of MII oocytes; (**C**) Rate of polar body extrusion. Data are expressed as mean ± SEM. Control, COCs and oocytes from healthy ovaries; PCO, COCs and oocytes from polycystic ovaries. *P < 0.05 and **P < 0.01, compared with control.

Polar body extrusion and parthenogenetic activation *in vitro* were employed to assess the developmental potential of oocytes. The COCs was well organized in control group, containing more than 5 peripheral granulosa cell layers, whereas in PCO group the COCs were not adequately expanded (Fig. 1A). Moreover, the oocytes in the PCO group showed significantly lower survival rate (Fig. 1B) and polar body extrusion rate (Fig. 1C), as compared with the control group.

No significant difference was observed between two groups in the cleavage rate after parthenogenetic activation at the 2-cell stage, yet the cleavage rate at the 4-cell stage (*P* < 0.05) and the blastocyst rate (*P* < 0.01) were significantly lower in PCO group compared with the control group. Cell numbers in blastocysts were also significantly reduced (*P* < 0.05) in the PCO group (Table 1).

### PCO oocytes demonstrate disrupted mitochondrial distribution

**Figure 2:**
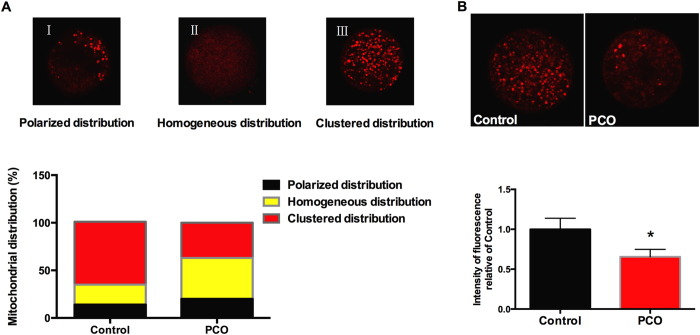
Distribution of mitochondria in oocytes. (**A**) Representative images and the ratios of different mitochondrial distribution patterns (magnification, × 400); (**B**) Fluorescent intensities of clustered mitochondria (magnification, × 400). Data are expressed as mean ± SEM. Control, oocytes from healthy ovaries; PCO, oocytes from polycystic ovaries. *P < 0.05 and **P < 0.01, compared with control.

Mitochondrial distribution pattern in GV oocytes was detected by using MitoTracker Red. Three distinct patterns, including polarized distribution, homogeneous distribution and clustered distribution, were seen (Fig. 2A). No significant differences were observed between two groups in the percentage of polarized and homogeneous distribution, yet the percentage of clustered mitochondria distribution was significantly lower (*P* < 0.05) in the PCO group compared with the control group (Fig. 2A), which was associated with significantly reduced (*P* < 0.05) fluorescent intensities (Fig. 2B).

### Decreased mitochondrial membrane potential and deformed mitochondrial ultrastructure are observed in PCO oocytes

**Figure 3:**
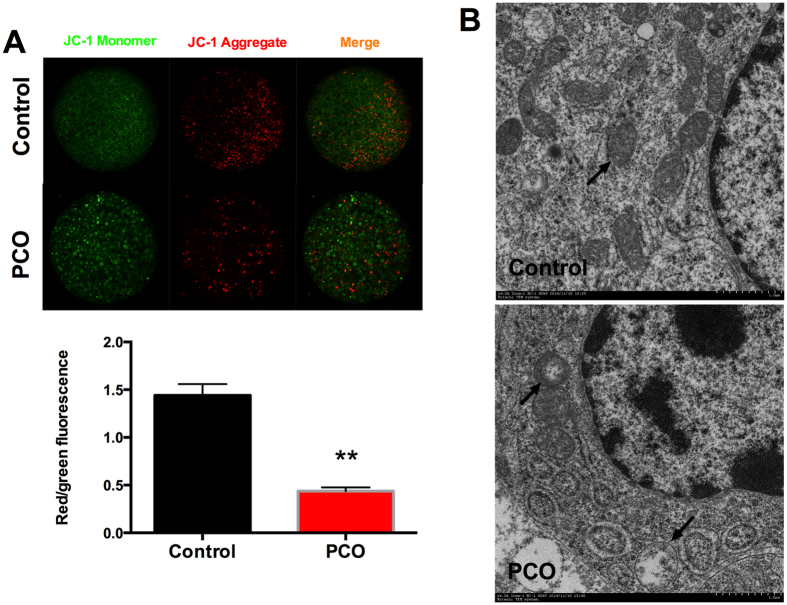
Mitochondrial membrane potential and mitochondrial ultrastructure. (**A**) JC-1 staining of oocytes (magnification, × 400) and the ratio of red to green fluorescence; (**B**) Transmission electron micrographs of the mitochondria (blank arrows) of control and PCO oocytes. Data are expressed as mean ± SEM. Control, oocytes from healthy ovaries; PCO, oocytes from polycystic ovaries. *P < 0.05 and **P < 0.01, compared with control.

Mitochondrial membrane potential was indicated by the ratio of red to green fluorescence in JC-1 staining. The red fluorescence was weaker and the green was stronger in the oocytes of PCO group, which led to significantly lower (*P* < 0.01) ratio of red to green fluorescence in PCO oocytes (Fig. 3A), indicating decreased mitochondrial membrane potential.

Transmission electron microscopy was employed to evaluate the changes of mitochondrial ultrastructure. Most of the mitochondria in control group contain clearly visible intact inner membrane, outer membrane, and a well-defined intermembrane space. In contrast, mitochondria in PCO group were predominantly spherical with almost no cristae (Fig. 3B).

### PCO oocytes show lower mtDNA copy number and down-regulated expression of mtDNA-encoded genes

**Figure 4:**
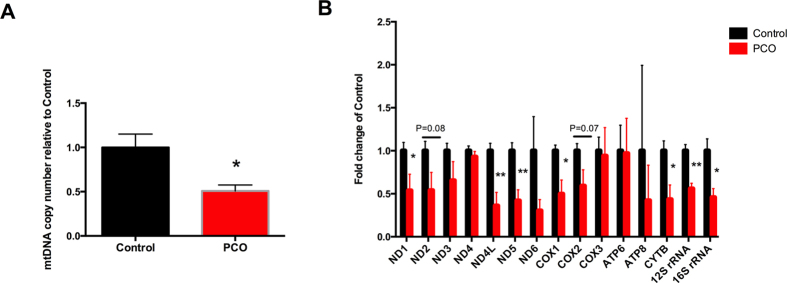
The mtDNA copy number and the expression of mtDNA-encoded genes in oocytes. (**A**) Relative mtDNA copy number; (**B**) Expression of mtDNA-encoded genes. Data are expressed as mean ± SEM. Control, oocytes from healthy ovaries; PCO, oocytes from polycystic ovaries. * *P* < 0.05 and ** *P* < 0.01, compared with control.

The copy number of mtDNA in the oocyte was significantly lower (*P* < 0.05) in PCO group than in control group (Fig. 4A). Meanwhile, 7 out of 13 mtDNA-encoded genes were down-regulated in PCO group (Fig. 4B). The mRNA abundance of *ND1, ND4L, ND5, COX1,* and *CYTB* was significantly lower in the oocytes of PCO group (*P* < 0.05); while that of *ND2* and *COX2* tended to be lower (*P* < 0.1). In addition, the abundance of *12S rRNA* and *16S rRNA* was also significantly lower (P < 0.05) in the oocytes of PCO group (Fig. 4B).

### Abnormally activated DNMT1 expression and one-carbon metabolic pathway are detected in PCO oocytes

**Figure 5:**
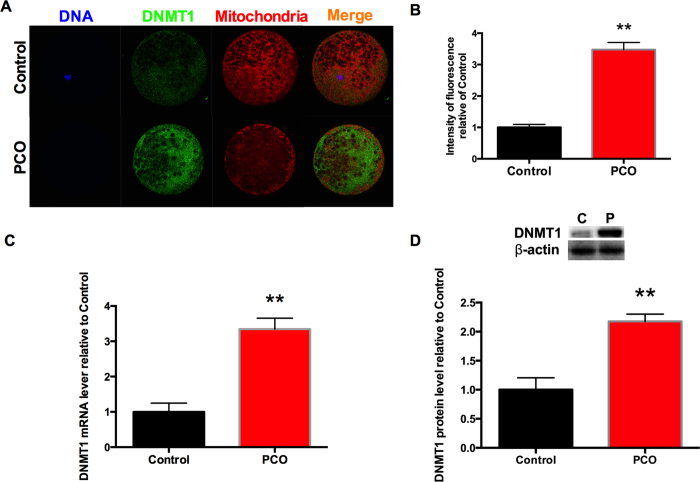
Expression and localization of DNMT1 in oocytes. (**A**) Immunofluorescent localization of DNMT1 protein (magnification, × 400); (**B**) Average DNMT1 fluorescent intensities; (**C**) Relative mRNA expression of DNMT1; (**D**) Protein content of DNMT1 in whole cell lysates. Data are expressed as mean ± SEM. Control or C, oocytes from healthy ovaries; PCO or P, oocytes from polycystic ovaries. *P < 0.05 and **P < 0.01, compared with control.

DNMT1, a DNA methyltransferases reported to target mitochondrion^47^, was examined by immunofluorescent staining. DNMT1 was translocated in the mitochondria (Fig. 5A). The fluorescence intensities of DNMT1 were significantly higher (*P* < 0.01) in PCO group (Fig. 5B), which was associated with significantly increased (*P* < 0.01) level of DNMT1 mRNA (Fig. 5C) and protein (Fig. 5D).

**Figure 6:**
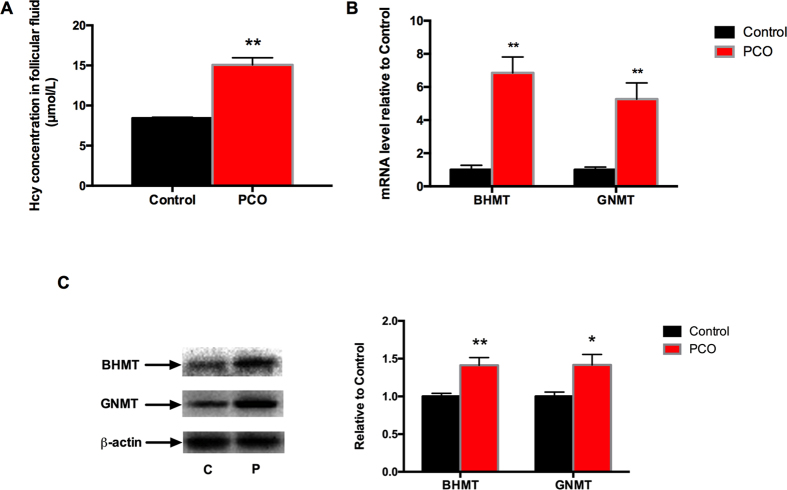
Concentration of Hcy and the expression of one-carbon metabolic enzymes. (**A**) Hcy concentration in follicular fluid; (**B**) The mRNA level of one-carbon metabolic enzymes; (**C**) The protein content of one-carbon metabolic enzymes. Data are expressed as mean ± SEM. Control or C, oocytes from healthy ovaries; PCO or P, oocytes from polycystic ovaries. *P < 0.05 and **P < 0.01, compared with control.

Moreover, the concentration of Hcy in follicular fluid was significantly higher (*P* < 0.01) in PCO group compared with the control group (Fig. 6A). Furthermore, BHMT and GNMT, which participate in one-carbon metabolic pathway, were significantly up-regulated (*P* < 0.05) at the level of both mRNA (Fig. 6B) and protein (Fig. 6C) in PCO group.

### mtDNA coding sequences are hypermethylated in PCO oocytes

**Figure 7:**
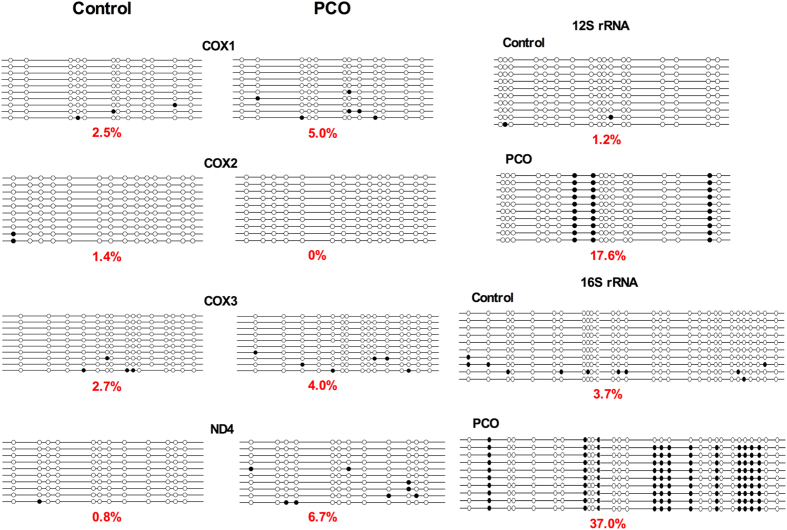
Methylation status of mtDNA sequences coding for *12S rRNA, 16S rRNA, COX1, COX2, COX3* and *ND4* genes. Control, oocytes from healthy ovaries; PCO, oocytes from polycystic ovaries. *P < 0.05 and **P < 0.01, compared with control.

The methylation status of mtDNA coding for *12S rRNA, 16S rRNA, COX1, COX2, COX3* and *ND4* was analyzed by bisulfite sequencing in oocytes of control and PCO groups. The mtDNA sequences coding for *12S rRNA* (*P* < 0.01), *16S rRNA* (*P* < 0.01) and *ND4* (*P* < 0.05) were significantly hypermethylated in PCO group (Fig. 7). The methylation status of the regulatory D-loop region was also analyzed (Figure S2). The amplified D-loop sequences are highly variable in length mainly due to different number of tandem repeats (492bp, 29 repeats)^48^, and also contain multiple single nucleotide polymorphisms (SNP). Regardless of all these variations, the percentage of methylated CpGs detected was significantly higher (*P* < 0.05) in PCO group (15.7%) compared with the control group (11.2%).

## Discussion

The present study provides the first evidence that the abnormal activation of one-carbon metabolism and the hypermethylation of mtDNA are associated with mitochondrial malfunction and the poor quality of oocytes derived from polycystic ovaries of gilts. Although the serum level of Hcy was not measured in the present study, the concentration of Hcy in the follicular fluid was significantly higher in polycystic ovaries. The elevated Hcy in the follicular fluid coincided with highly activated one-carbon metabolic pathway, up-regulated DNMT1 expression, as well as mtDNA hypermethylation. These metabolic and epigenetic changes may account for the disrupted mitochondrial function and thus the deteriorated oocyte quality in PCOS gilts. These results may help to understand the mechanisms underlying the link between decreased oocyte quality and hyperhomocysteinemia in PCOS patients.

It has been well documented that oocytes from PCOS patients are of poor quality, with impaired competence for future fertilization^3^,^4^,^5^,^6^,^7^. Although PCOS women produce more oocytes and more embryos in *in vitro* fertilization, they still suffer from lower pregnancy rate^5^,^49^,^50^ and higher miscarriage rate^9^,^10^. Similarly, decreased developmental potential of oocytes and resulting embryos has been reported in a mouse PCOS model induced by dehydroepiandrosterone (DHEA)^51^. In this study, we show, for the first time, that the polycystic ovaries of gilts also produce poor quality oocytes with impaired developmental potential indicated by lower survival rate, polar body extrusion rate and cleavage rate.

It is well known that mitochondria, the organelles responsible for energy production, are essential for optimal oocyte maturation, fertilization and embryonic development^52^. Mitochondrial dysfunction leads to poor oocyte quality^22^,^23^ and compromised embryonic development^24^. High quality oocytes contain optimal numbers of mitochondria and produce sufficient levels of ATP^53^ required for developing high quality blastocysts after fertilization^54^. Low ATP and decreased mtDNA copy number are associated with not only the poor oocyte quality, but also the retarded embryo development and suboptimal implantation and placentation rates^53^,^55^,^56^. Mitochondria dysfunction is observed in the oocytes retrieved from DHEA-induced mouse PCOS model, which is indicated by lower mtDNA copy number and ATP content, decreased inner mitochondrial membrane potential, excessive oxidative stress and impaired embryo development competence^51^. In agreement with these observations, oocytes retrieved from the polycystic ovaries of the gilts also demonstrated disrupted mitochondrial distribution pattern, decreased mitochondrial membrane potential and deformed mitochondrial ultrastructure, which was associated with lower mtDNA copy number and diminished expression of mtDNA-encoded genes.

Hcy is a non-essential, sulfur-containing amino acid formed during the metabolism of methionine. A number of studies reported elevated Hcy concentration in the serum^18^,^19^,^20^ and follicular fluid^21^ of PCOS patients. Higher Hcy concentration in follicular fluid is associated with poor oocyte and embryo qualities in PCOS patients undergoing assisted reproduction^21^. Same phenomenon is observed in the present study in polycystic ovaries of gilts, yet the reason for elevated Hcy in PCOS remains unclear. The homeostasis of Hcy is maintained by two primary pathways, namely the remethylation pathway, where Hcy is converted back to methionine, and the transsulfuration pathway, where Hcy is converted to cystathionine and then to cysteine^57^. Elevated Hcy level in follicular fluid could be primary due to deregulated Hcy metabolism in the ovary or secondary to higher serum Hcy level owing to disrupted Hcy metabolism in organs other than ovary. The latter appears more likely the case, because BHMT, a betaine homocysteine methyltransferase that converts Hcy to methionine, was actually up-regulated, probably, in response to elevated Hcy. Also, GNMT, the glycine N-methyltransferase that transforms S-Adenosyl methionine (SAM) to S-adenosylhomocysteine (SAH) and thereby donates a methyl group, and DNMT1, the DNA (cytosine-5-)-methyltransferase 1 that transfers the released methyl group to CpG residues of target DNAs, were both up-regulated in oocytes derived from polycystic ovaries of gilts, indicating abnormally activated one-carbon metabolic pathway and modified DNA methylation process.

To our knowledge, there is no explanation how high Hcy contributes to poor oocyte quality. It is reported that Hcy competes with methionine for uptake into the embryo, and thus hinders the formation of SAM, the universal methyl donor for DNA and/or protein methylation^58^. Although Hcy is considered to act as a methylation inhibitor, the correlation between Hcy and DNA methylation is inconsistent. For instance, a negative correlation of plasma Hcy levels with lymphocyte DNA methylation was reported in healthy young women^40^, while a positive correlation of plasma Hcy with global DNA methylation in peripheral lymphocytes was observed in the coronary artery disease patients^59^. It appears that the correlation between Hcy and global DNA methylation is dependent on the range of endogenous Hcy levels. Negative correlation is observed when Hcy is within the physiological range, while positive correlation occurs when Hcy level is abnormally high in diseased state. While previous studies mainly focused on the association of Hcy with the methylation status of global genomic DNA^60^ or the promoter of candidate nuclear-encoded genes^61^, we show here a positive correlation between elevated Hcy concentration in follicular fluid and mtDNA hypermethylation in the oocytes of PCO gilts.

Recently, growing interests are directed towards the mtDNA methylation and its impact on health and disease, despite a long debate over the existence of mtDNA methylation^62^. Similar to nuclear DNA methylation, deregulated mtDNA methylation has been found to be associated with disease development^30^,^31^,^63^, adverse environmental exposure^32^,^33^,^34^, drug treatment^35^, and aging^36^. An isoform of DNMT1 has been found to specifically target mitochondria to methylate mtDNA and finally modifies transcription of the mitochondrial genome^47^. Some DNMT1 mutations are linked with DNMT1 abnormal activity and mitochondrial dysfunction^62^. In our study, mtDNA hypermethylation in the oocytes of PCO gilts was associated with a significantly higher immunofluorescent staining of DNMT1 in the mitochondria and an up-regulation of DNMT1 expression at both mRNA and protein levels.

The displacement loop (D-loop) in the mitochondrial genome contains promoters for mtDNA replication or transcription, and the methylation level in this control region manifests an important role in modulating either replication or transcription of mtDNA^64^. Therefore, the epigenetic regulations of the D-loop and its implications in health and disease have been a focus of a number of studies^63^,^65^. For instance, a correlation between D-loop methylation and expression of *ND2*, a subunit of NADH encoded by mtDNA, was observed in tumor and corresponding noncancerous tissues from colorectal cancer patients^30^. In addition, the methylation status of *ND6*, which is another crucial subunit for complex I assembly, significantly impacts the transcriptional regulation of the gene in the liver of nonalcoholic steatohepatitis^31^. Recently, the status of mtDNA methylation was investigated in workers exposed to airborne pollutants^34^. *12S rRNA* coding region was found to be hypermethylated in exposed subjects, and an inverse correlation was observed between placental mtDNA methylation and mtDNA content^33^.

In this study, we examined the methylation status of mtDNA sequences coding for *12S rRNA, 16S rRNA, COX1, COX2, COX3* and *ND4*, as well as the regulatory D-loop region. *12S rRNA, 16S rRNA* and *ND4* coding sequences were hypermethylated in PCO oocytes, which is in accordance with suppressed expression of mtDNA-encoded genes and impaired mitochondrial function. Despite the highly variable length of the amplicons due to different numbers of tandem repeats and a number of random SNPs or non-CpG methylations in the regulatory D-loop region, the percentage of methylated CpGs detected was significantly higher in PCO oocytes. The hypermethylation of the D-loop region was associated with lower mtDNA content and down-regulated expression of mtDNA-encoded genes, implicating possible role of mtDNA methylation on the replication and transcription of mitochondrial genome.

Although the data presented here are primarily descriptive and the proposed link between high Hcy and poor oocyte quality is deduced mainly from association analysis, the findings may shed a new light on the pathogenesis of PCOS and provide new hints for identifying novel targets for the prevention and treatment of this disease.

## Additional Information

**How to cite this article**: Jia, L. *et al.* Abnormally activated one-carbon metabolic pathway is associated with mtDNA hypermethylation and mitochondrial malfunction in the oocytes of polycystic gilt ovaries. *Sci. Rep.* **6**, 19436; doi: 10.1038/srep19436 (2016).

## Supplementary Material

Supplementary Information

## Figures and Tables

**Table 1:** Developmental competence of parthenogenetically activated embryos.

Parameters	Control	PCO
Total PA oocytes	163	130
Number of 2- cell(%)	155(95.11 ± 0.48)	115(93.89 ± 0.56)
Number of 4-cell(%)	134(82.30 ± 0.56)	95(76.12 ± 0.65)*
Number of Blastocyst(%)	84(51.68 ± 1.85)	26(29.15 ± 3.62)**
Totle cell number per blastocyst	40.66 ± 2.33	31.67 ± 1.76*

Data are expressed as mean ± SEM. Control, oocytes from healthy ovaries; PCO, oocytes from PCO. *P < 0.05 and **P < 0.01, compared with control.
