# Supplementary material for: Abnormally activated one-carbon metabolic pathway is associated with mtDNA hypermethylation and mitochondrial malfunction in the oocytes of polycystic gilt ovaries
Source: Sci Rep. 2016 Jan 13;6:19436. doi: 10.1038/srep19436 (PMC4725837; doi:10.1038/srep19436)
Supplement: Supplementary Information [file srep19436-s1.doc]

**Abnormally activated one-carbon metabolic pathway is associated with mtDNA hypermethylation and mitochondrial malfunction in the oocytes of polycystic gilt ovaries**

Longfei Jia^1^, Juan Li^2^, Bin He^1^, Yimin Jia^1^, Yingjie Niu^2^, Chenfei Wang^2^, Ruqian Zhao^1, 3*^

**Supplemental Figure S1**


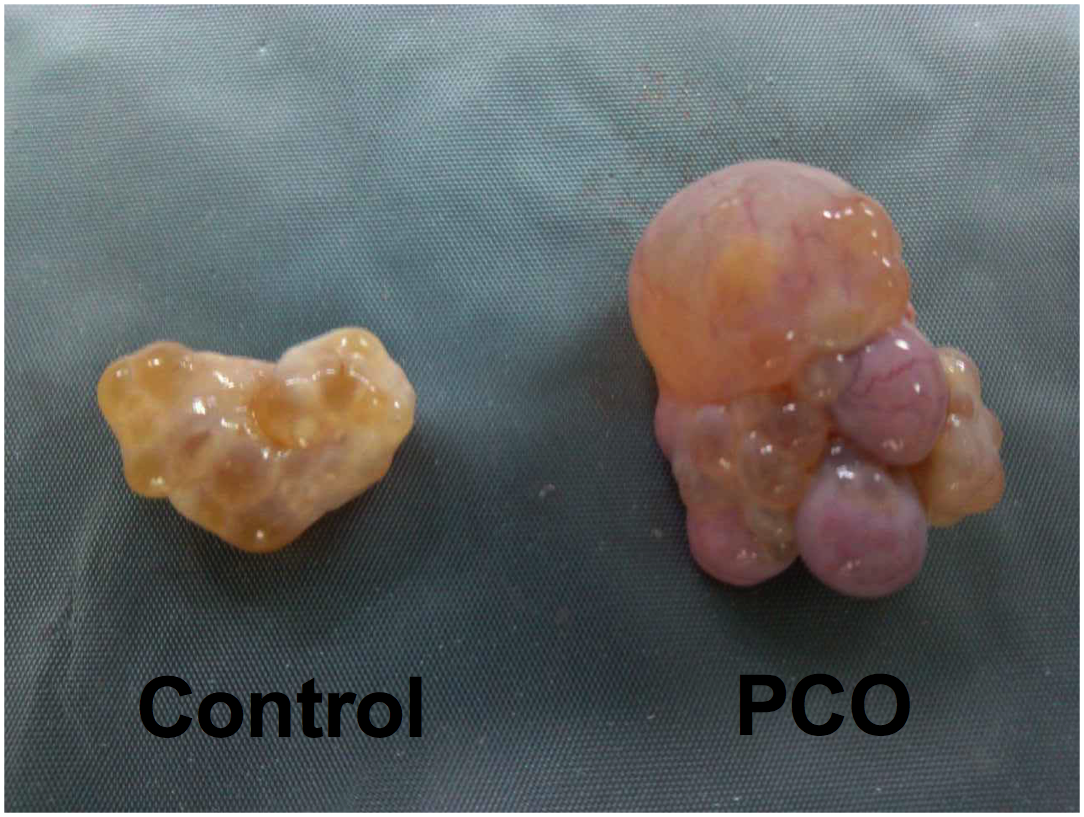


**Figure S1** Representative images of healthy and polycystic ovaries

**Supplemental Figure S2**


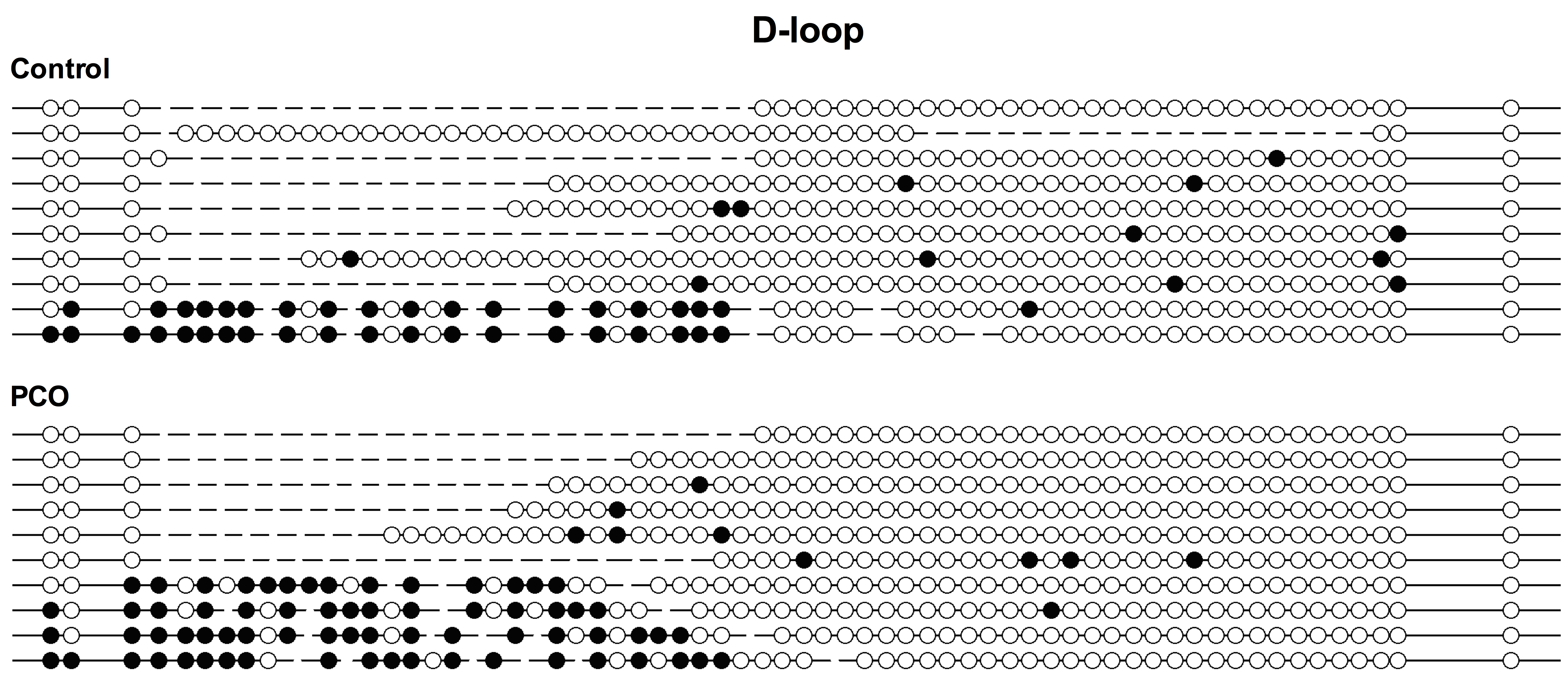


**Figure S2** Methylation status of displacement loop (D-loop) region. Control, oocytes from healthy ovaries; PCO, oocytes from polycystic ovaries.

**Supplemental Table S1** Nucleotide sequences of primers

| Target genes | Sequences (5’ to 3’) |  |
| --- | --- | --- |
| mRNA expression |  |  |
| *DNMT1* | F:TCAGGGACCACACTGTAAG | R:GCTGCAGCCATTCTTCTTGT |
| *BHMT* | F:GAATGTCATGCAGACCTTCACC | R:CTTCCGTTTCGCTCTTGC |
| *GNMT* | F:ACATGGTGACCCTCGATTACAC | R:CCTCACTCAGTCCGTCTTCCT |
| *MAT2b* | F:CTGACAGTCCTGTCTTGGGAGC | R:GCCAGAGTGATTCTTTGATGCC |
| *AHCYL1* | F:GTGGTGGTGTGTGGCTACGG | R:GCAGAGCACAGATGGGGTCA |
| *COX1* | F:TGGTGCCTGAGCAGGAATAGTG | R:ATCATCGCCAAGTAGGGTTCCG |
| *COX2* | F:GCTTCCAAGACGCCACTTCAC | R:TGGGCATCCATTGTGCTAGTGT |
| *COX3* | F:GGCTACAGGGTTTCACGGGTTG | R:TCAGTATCAGGCTGCGGCTTCA |
| *ND3* | F:AGCACGCCTCCCATTCTCAAT | R:TGCTAGGCTTGCTGCTAGTAGG |
| *CYTB* | F:CTGAGGAGCTACGGTCATCACA | R:GCTGCGAGGGCGGTAATGAT |
| *ND1* | F:TCCTACTGGCCGTAGCATTCCT | R:TTGAGGATGTGGCTGGTCGTAG |
| *ND2* | F:ATCGGAGGGTGAGGAGGGCTAA | R:GTTGTGGTTGCTGAGCTGTGGA |
| *ND4L* | F:GATCGCCCTTGCAGGGTTACTT | R:CTAGTGCAGCTTCGCAGGCT |
| *ND4* | F:TCGCCTATTCATCAGTAAGTCA | R:GGATTATGGTTCGGCTGTGTA |
| *ND5* | F:CGGATGAGAAGGCGTAGGAA | R:GCGGTTGTATAGGATTGCTTGT |
| *ND6* | F:ACTGCTATGGCTACTGAGATGT | R:CTTCCTCTTCCTTCAACGCATA |
| *ATP6* | F:ACTCATTCACACCCACCACACA | R:CCTGCTGTAATGTTGGCTGTCA |
| *ATP8* | F:TGCCACAACTAGATACATCC | R:GCTTGCTGGGTATGAGTAG |
| *PPIA* | F:GACTGAGTGGTTGGATGG | R:TGATCTTCTTGCTGGTCTT |
| *18S* | F:CCCACGGAATCGAGAAAGAG | R:TTGACGGAAGGGCACCA |
| Bisulfite sequencing | |  |
| *12S rRNA* | F:GGTTTGGTTTTGGTTTTTTTATTAAT | R:CAAATCCTTTAAATTTTAAACAATTAC |
| *16S rRNA-1* | F:GGTTTAAAAGTAGTTATTAATTGAGAAAG | R:AAAAATAAAAAACAATAAAACCCTC |
| *16S rRNA-2* | F:GAGGGTTTTATTGTTTTTTATTTTTAATTA | R:ATTAATCCCATTTCTCTTATCCTTTC |
| *COX1* | F:TTTTGATTTAGTAGGTGGTGGAGAT | R:AAAAAAAATCAATAAACAAAACCCC |
| COX2 | F:AAATTGATATATATTAGTATAATGGATGTT | R:TCCACAAATTTCTAAACACTATCC |
| COX3 | F:TTGTTTTTTATTGGATTTTTTTGAGTT | R:AAAATCCTCATCAATAAATTAATAC |
| *ND4* | F:TAGTTTTTATAGTAAAAATATTTTTGTA | R:ATTTATAAAACTTACTACTAATCATCATAT |
| *D-LOOP* | F:TTGGATTTTATGGAATTTATGATT | R:TACACTCTACTTTATTTTTAAAATTTAACA |
